# The technical landscape of modern epilepsy surgery

**DOI:** 10.3171/2024.4.FOCVID23219

**Published:** 2024-07-01

**Authors:** Sharona Ben-Haim, Dennis Spencer, Jonathan Roth, Howard L. Weiner

**Affiliations:** 1 Department of Neurological Surgery, University of California, San Diego, California;; 2 Department of Neurosurgery, Yale University School of Medicine, New Haven, Connecticut;; Departments of 3 Neurosurgery and; 4 Pediatric Neurosurgery, Tel Aviv Medical Center, Tel Aviv University, Tel Aviv, Israel;; 5 Division of Pediatric Neurosurgery, Department of Surgery, Texas Children’s Hospital, Houston, Texas; and; 6 Department of Neurosurgery, Baylor College of Medicine, Houston, Texas

The landscape of epilepsy surgery has expanded dramatically over the last 25 years, reflecting both philosophic and technical advances. These exciting developments have enabled the possibility of this surgical option for many more patients than previously thought and have been made possible by progress in both traditional and novel diagnostic and treatment approaches. Whereas previously only patients with concordant lesional or focus-dominant refractory epilepsy were considered for surgery, now patients with more complex network-dominant pathologies and multifocal, or even generalized, epilepsy are potential surgical candidates. In addition to this philosophic advance, there has been a parallel evolution in epilepsy surgery techniques, with the widespread shift from the use of subdural grids to the minimally invasive stereoelectroencephalography (SEEG) as the universally accepted method for invasive intracranial monitoring. Similarly, whereas previously resection and/or disconnection of the seizure focus was the traditional therapeutic intervention in epilepsy surgery, currently patients have the option of undergoing thermoablation of the surgical target with stereotactic laser interstitial thermal therapy (LITT) or radiofrequency (RF) ablation, and neuromodulation of the brain with vagus nerve stimulation (VNS), deep brain stimulation (DBS), and responsive neurostimulation (RNS). Treatment is individualized and tailored to the specific patient.

The modern epilepsy neurosurgeon is a master of both traditional, open, craniotomy-based operative techniques and an array of minimally invasive stereotactic approaches. Here, we highlight several video demonstrations of paradigmatic operative procedures in epilepsy surgery, both traditional and novel, since both approaches are critically important in the neurosurgeon’s armamentarium: VNS placement, microsurgical resection of epileptogenic lesions, temporal lobectomy including novel approaches to the mesial temporal lobe, hemispherotomy, SEEG, DBS, RNS, LITT, and RF thermocoagulation. In the modern era, the importance of collaboration in epilepsy surgery and the role of the surgical team needs to be stressed. Beyond the collaboration between neurosurgery, neurology, radiology, and other technological and research specialists, there is also a role for collaboration between surgeons. The ability of a single surgeon to master all techniques at the highest level, as apparent in this array of technical videos, may not be as advantageous as the technical collaboration of different surgeons with expertise in the various individual procedures. This will enable focused expertise and will eventually benefit the patient greatly. The technical videos presented here will facilitate a better understanding of the wide range of both traditional and novel approaches in treating patients with medically refractory epilepsy.

## Disclosures

The authors report no conflict of interest concerning the materials or methods used in this study or the findings specified in this publication.

